# A Comprehensive and Critical Evaluation of Autonomic Nervous System Impairment in Patients with Wilson Disease: A Cross‐Sectional Study

**DOI:** 10.1002/brb3.71624

**Published:** 2026-08-10

**Authors:** Sabyasachi Pattanayak, Niraj Kumar Srivastava, Anand Kumar, Deepika Joshi, Atanu Roy, Sanjeev Kumar Singh, Rajniti Prasad, Suyash Tripathi, Ashish Verma, Ritu Ojha, Varun Kumar Singh, Abhishek Pathak, Rameshwar Nath Chaurasia, Vijaya Nath Mishra, Ibrahim Hussain

**Affiliations:** ^1^ Department of Neurology, Institute of Medical Sciences Banaras Hindu University Varanasi India; ^2^ Department of Physiology, Institute of Medical Sciences Banaras Hindu University Varanasi India; ^3^ Department of Pediatrics, Institute of Medical Sciences Banaras Hindu University Varanasi India; ^4^ Department of Cardiology, Institute of Medical Sciences Banaras Hindu University Varanasi India; ^5^ Department of Radiodiagnosis and Imaging Institute of Medical Sciences Banaras Hindu University Varanasi India

**Keywords:** autonomic impairment, autonomic nervous system (ANS), autonomic testing, cardiovascular testing, copper metabolism disorder, neurodegenerative disorders, neurological manifestations, sympathetic and parasympathetic dysfunction, Wilson's disease

## Abstract

**Background:**

Wilson's disease (WD) is an autosomal recessive disorder of copper metabolism with heterogeneous hepatic and neurological manifestations. Autonomic nervous system (ANS) involvement in WD is poorly recognized and remains inadequately studied, despite its potential to cause significant morbidity if unrecognized. This study aimed to assess the presence and severity of autonomic dysfunction in patients with WD and to examine its association with clinical and biochemical parameters using standardized autonomic tests and comprehensive cardiac evaluation.

**Methods:**

A comparative cross‐sectional study was conducted at a tertiary care center between September 2021 and March 2023. Patients with a confirmed diagnosis of WD, based on clinical, biochemical, and genetic criteria, were enrolled and compared with age‐ and sex‐matched healthy controls. All participants underwent biochemical evaluation, including serum ceruloplasmin, 24‐h urinary copper excretion, and liver function tests. Autonomic function was assessed using Ewing's battery of tests (deep breathing, Valsalva maneuver, head‐up tilt [HUT], isometric handgrip and cold pressor test)and heart rate variability (HRV) analysis. Cardiac assessment included transthoracic echocardiography, 24‐h Holter monitoring, and cardiac magnetic resonance imaging. Statistical analysis comprised descriptive statistics and Kendall's correlation analysis, with statistical significance set at *p* < 0.05.

**Results:**

Patients with WD demonstrated significant autonomic dysfunction compared with healthy controls, predominantly involving parasympathetic impairment, as evidenced by abnormal Ewing's test responses and reduced HRV indices. Although no overt structural cardiac abnormalities were identified, cardiac rhythm disturbances were observed in a subset of WD patients.

**Conclusion:**

Autonomic dysfunction, particularly parasympathetic impairment, is a clinically relevant but underrecognized feature of WD. Incorporating systematic autonomic function assessment into routine clinical evaluation may enable early detection of subclinical dysfunction, guide timely interventions, and potentially prevent progression to clinically significant autonomic complications in patients with WD.

## Graphical abstract

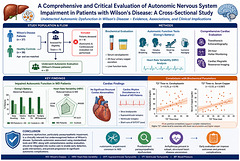

**Abbreviations**
∆DBP: change in diastolic blood pressure
∆HR: change in heart rate
AVG rate: average rate
CAN: cardiovascular autonomic neuropathy
DBT: deep breathing test
DM: diabetes mellitus
E/A: early wave to late wave ratio
HF: heart failure
HRT: heart rate turbulence
HRV analysis: heart rate variability
HUT: head‐up tilt
LF/HF ratio: low‐frequency/high‐frequency ratio
LV function: left ventricle function
MVA: mitral valve area
OPD: outpatient department
PHT: pulmonary half time
RMSSD: root mean square of successive differences between heartbeats
RVSP: right ventricle systolic pressure
SDRR: standard deviation of R‐R intervals
UWDRS: Unified Wilson's Disease Rating Scale
VR: Valsalva ratio
WD: Wilson's disease

## 1 Introduction

Wilson's disease (WD) is a rare autosomal recessive disorder caused by mutations in the ATP7B gene, resulting in defective copper excretion and reduced incorporation into ceruloplasmin. Its prevalence ranges from 1:30,000 to 1:100,000, with higher rates reported in certain populations (Lafhal et al. 2023; Deguti et al. 2004). Copper accumulation initially occurs in the liver and subsequently affects the brain, particularly the basal ganglia, leading to hepatic manifestations such as hepatitis, cirrhosis, and acute liver failure, as well as neurological features including tremor, rigidity, dystonia, and dysarthria (Biswas et al. 2017; Yuan et al. 2021). The clinical presentation of WD is highly variable, with disease onset typically occurring between 5 and 35 years of age and involving hepatic, neurological, or psychiatric symptoms (Lafhal et al. 2023; Deguti et al. 2004; Biswas et al. 2017; Yuan et al. 2021). Treatment primarily focuses on reducing systemic copper levels through chelating agents such as penicillamine and trientine or through zinc salts, and lifelong therapy is essential for optimal disease management (Lafhal et al. 2023; Yuan et al. 2021).

Neurological involvement in WD affects several critical brain regions, including the thalamus, hypothalamus, basal ganglia (globus pallidus, putamen, and caudate nucleus), and brainstem structures such as the midbrain, pons, and medulla, all of which play important roles in autonomic nervous system (ANS) regulation (Deguchi et al. 2005). Damage to these areas can disrupt both motor and autonomic pathways, resulting in symptoms such as orthostatic hypotension, urinary dysfunction, and impaired thermoregulation (Ala et al. 2007; Li et al. 2015). Autonomic dysfunction in WD is predominantly recognized to excessive copper accumulation within the central autonomic network, particularly involving the basal ganglia, hypothalamus, thalamic connections, and brainstem autonomic nuclei. Copper‐induced neurotoxicity within these regions disrupts autonomic integration and neurophysiological regulation, leading to widespread disturbances in cardiovascular, gastrointestinal, sudomotor, and thermoregulatory functions. In contrast to Parkinson's disease, where autonomic impairment is largely driven by peripheral postganglionic sympathetic denervation secondary to α‐synuclein‐mediated neurodegeneration, the autonomic abnormalities observed in WD primarily reflect central autonomic dysregulation rather than peripheral autonomic failure. This distinction is crucial because, regardless of occasional overlap in autonomic testing profiles, such as abnormalities in heart rate variability (HRV), sympathetic skin response, or cardiovascular reflex testing are fundamentally different. Therefore, similarities in autonomic manifestations between WD and Parkinson's disease should not be misconstrued as evidence of shared neurodegenerative pathways but rather interpreted within the distinct context of copper‐mediated central neuronal dysfunction unique to WD (Kłysz et al. 2021; Li et al. 2017; Cersosimo and Benarroch 2013; Bandmann et al. 2015; Machado et al. 2006; Coon et al. 2018; Palma and Kaufmann 2018; Jain 2011; Leys et al. 2022; Li et al. 2015).

Previous investigations into autonomic dysfunction in WD have produced inconsistent findings (Meenakshi‐Sundaram et al. 2002). Although some patients exhibit prominent autonomic symptoms, many do not demonstrate significant peripheral nervous system involvement, suggesting that autonomic dysfunction in WD is largely of central origin (Chu et al. 1997; Bhattacharya et al. 2002). Notably, autonomic symptoms often improve soon after initiation of chelation or targeted therapy, paralleling early neurological and hepatic recovery (Li et al. 2017; Joshi et al. 2023). Studies by Deguchi et al. (2005), Kumar (2005), and Brewer et al. (1998) also reported significant improvement in autonomic function following treatment. However, the long‐term course of autonomic dysfunction during therapy remains unclear, emphasizing the need for further longitudinal studies (European Association for the Study of the Liver 2012).

Several advanced methods are used to evaluate cardiovascular autonomic neuropathy (CAN), particularly in diabetes mellitus (DM), including the Ewing test battery, HRV, and heart rate turbulence (HRT) analysis (Li et al. 2017). The Ewing battery remains the gold standard because of its high sensitivity and specificity, although it is relatively time‐consuming and requires specific preparation. It consists of five principal tests: heart rate response to standing (30:15 ratio), the Valsalva maneuver, deep breathing–induced respiratory sinus arrhythmia, blood pressure response to the cold pressor test, and sustained handgrip. These tests have well‐established normative values that allow classification of autonomic function as normal, early abnormal, or definite neuropathy according to standard guidelines (Granowetter 1995). In routine clinical practice, however, performing the complete Ewing test battery may be difficult due to time constraints, limited resources, or poor patient compliance. To address this limitation, the present study evaluates the predictive value of individual autonomic tests and their combinations, aiming to identify subsets with optimal diagnostic performance under different cost‐function constraints. The study also presents results for all test combinations and provides graphical tools to assist clinical decision‐making (Cheshire et al. 2021).

HRV, recommended by the American Diabetes Association, is a useful non‐invasive measure of autonomic function; however, its interpretation may be influenced by factors such as age, medications, comorbidities, circadian rhythms, and acute stress (Ooi et al. 2024). In WD, cardiac involvement has historically received less attention than hepatic or neurological manifestations, although case reports describing sudden cardiac death, arrhythmias, and unrecognized dilated cardiomyopathy suggest potential cardiac risks (Brewer et al. 1998; Deguchi et al. 2005). A significant longitudinal study by Grandis et al. (2017) demonstrated an increased risk of heart failure (HF) and atrial fibrillation in WD patients, challenging the previous notion that cardiac involvement is benign. These findings suggest that copper accumulation may contribute to myocardial dysfunction and electrical instability, although detailed mechanistic evidence remains limited. Identifying WD patients at increased cardiovascular risk is therefore essential. Early detection of cardiac autonomic dysfunction may facilitate timely preventive strategies and closer clinical monitoring. Consequently, the present study was designed to systematically evaluate autonomic dysfunction in patients with WD, with particular emphasis on cardiac autonomic involvement associated with copper overload. In addition, the study aims to investigate the relationship between autonomic function indices and biochemical markers of copper metabolism in order to better characterize the cardiovascular risks linked to impaired copper homeostasis in WD.

## 2 Materials and Methods

### 2.1 Study Design

The present study was designed as a case‐controlled observational investigation, aimed at systematically assessing the presence and extent of autonomic dysfunction in individuals diagnosed with WD in comparison to a carefully selected control group of healthy subjects. By adopting an observational design, the research allowed for the natural assessment of autonomic function parameters as they occurred in real‐life clinical settings, without the introduction of any experimental interventions or manipulations. This approach provided the opportunity to explore and document the inherent differences in ANS performance between patients affected by WD and healthy individuals, thereby reflecting true physiological and pathological variations. The case group was composed of patients who had been clinically and diagnostically confirmed to have WD, based on established diagnostic criteria, including biochemical tests and genetic analysis. These patients represented the target population in which autonomic dysfunction was suspected to be present due to the known neurodegenerative and hepatic involvement in WD. In parallel, the control group was carefully assembled by selecting healthy individuals who were matched to the cases in terms of key demographic characteristics, specifically age and gender, to minimize confounding factors and allow for a meaningful comparison of autonomic function between the two groups. This matching ensured that any observed differences could be more confidently attributed to the presence of WD, rather than variations in age or gender distribution.

### 2.2 Study Duration

The research was meticulously conducted over an extended period, spanning from September 2021 to March 2023. During this timeframe, a systematic and comprehensive approach was adopted to ensure thorough data collection, careful observation, and rigorous analysis. Each phase of the study was executed in accordance with the predefined research protocols, allowing for the collection of consistent and reliable information throughout the study period.

### 2.3 Study Area

The research was methodically carried out in the Department of Neurology, which is part of a renowned university teaching hospital known for providing advanced and specialized medical care. This esteemed institution not only serves as a center for high‐quality patient treatment but also emphasizes medical education and cutting‐edge research, creating an ideal environment for conducting rigorous scientific investigations in the field of neurology.

### 2.4 Study Participants

The research was conducted in the Department of Neurology at a tertiary care university teaching hospital, which is equipped to provide advanced medical care and specialized services in the field of neurology. On the basis of previous studies and 5‐year patient data from our hospital, the sample size was calculated assuming a moderate‐to‐large effect size (correlation coefficient, *r* = 0.46), with a statistical power of 80% and a two‐sided alpha error of 5%. Therefore, the minimum required sample size was estimated to be 35 participants. Considering WD to be a rare disease, all consecutive eligible patients during the study period were recruited. A total of 37 individuals diagnosed with WD were systematically recruited for participation in this study. The study population consisted of patients who were either admitted to the neurology ward or regularly attending the neurology/movement disorder outpatient department (OPD) at the tertiary care center. To ensure a consistent and adequate observation period, only those patients who had received continuous neurological care at the facility for at least one full year were considered eligible. Prior to enrollment, a thorough screening process was implemented to apply strict exclusion criteria and ensure the validity of the study. Patients with any pre‐existing cardiac abnormalities, other systemic medical conditions known to cause autonomic dysfunction, or with decompensated cirrhosis were systematically excluded from the study cohort to eliminate potential confounding factors that could affect autonomic function independently of WD. The diagnosis of WD in the recruited patients was confirmed by applying the standardized and internationally recognized scoring system developed during the eighth International Meeting on WD and Menke's Disease. This comprehensive scoring system incorporates clinical, biochemical, and genetic parameters to establish a reliable diagnosis of WD. For comparative analysis, a control group of 38 healthy individuals was recruited. These controls were carefully matched to the patient group in terms of age and sex to minimize demographic biases. In addition, all control participants were rigorously evaluated to ensure the absence of any ANS abnormalities, thereby serving as a baseline reference for assessing the specific autonomic dysfunction associated with WD.

### 2.5 Data Collection and Procedure

A comprehensive and systematic data collection process was initiated following the acquisition of informed consent from all participants. This process began with a detailed patient evaluation, encompassing a thorough medical history and a meticulous neurological examination. Subsequently, each patient underwent a series of standardized biochemical investigations, which included complete blood count (CBC), measurement of lactate dehydrogenase (LDH), assessment of serum copper levels, serum ceruloplasmin quantification, and a 24‐h urinary copper excretion test to assess copper metabolism and excretion. Additionally, a specialized ophthalmological assessment was performed to detect the presence of Kayser–Fleischer (KF) rings using slit‐lamp examination, a hallmark feature in WD. For clinical assessment of neurological and autonomic involvement, the Unified Wilson's Disease Rating Scale (UWDRS) was applied to evaluate the extent of neurological disability, and the Compass‐31 questionnaire was administered to assess the degree of autonomic symptoms subjectively. Autonomic function was evaluated using two specific methodologies: HRV analysis and Ewing's battery of cardiovascular reflex tests, both conducted under controlled laboratory settings by the Department of Physiology. Participants were instructed to abstain from caffeine‐containing beverages, including tea and coffee, for at least 24 h prior to autonomic assessment. To minimize the potential influence of postprandial metabolic variations, subjects were advised to consume only a light meal at least 2 h before testing. In addition, all participants were requested to maintain a minimum of 8 h of uninterrupted nocturnal sleep on the night preceding the evaluation. All autonomic function tests were conducted during the morning hours in a quiet, temperature‐controlled laboratory environment maintained between 22°C and 25°C, thereby reducing the potential confounding effects of environmental stimuli and circadian fluctuations on autonomic indices. For HRV analysis, artifact‐free Lead II electrocardiographic (ECG) recordings were acquired from all participants in the resting supine position following an adequate period of acclimatization. During the recording session, subjects were instructed to remain relaxed, awake, and motionless while avoiding unnecessary physical movement or conversation to minimize recording artifacts and autonomic perturbations. ECG signals were digitally acquired using a 4‐channel Power Lab data acquisition system (AD Instruments, Australia) at a sampling frequency of 1024 Hz, ensuring high temporal resolution for precise HRV analysis. The Power Lab data acquisition system was employed to collect precise physiological signals, which were subsequently analyzed using LabChart software to quantify autonomic responses. Ewing's battery consisted of a comprehensive set of autonomic reactivity tests, designed to assess both parasympathetic and sympathetic nervous system function. The complete Ewing's battery could be performed only in a subset of patients (21 patients), as several participants were unable to complete the full autonomic assessment because of severe dystonia, tremor, impaired motor coordination, fatigue, poor tolerance to prolonged testing procedures, and logistic limitations encountered during the study period. Importantly, patients with incomplete testing did not differ significantly from the rest of the cohort in terms of age, sex distribution, or overall disease severity, thereby reducing the likelihood of substantial selection bias in the analyzed data.

These included the deep breathing test (DBT) (assessing respiratory sinus arrhythmia), Valsalva maneuver (evaluating bar reflex function), isometric handgrip test (measuring sympathetic response to sustained muscle contraction), cold pressor test (eliciting sympathetic activation through cold‐induced stress), and the lying‐to‐standing test or head‐up tilt (HUT) test (assessing orthostatic cardiovascular reflexes). HUT testing was performed using a standardized passive tilt‐table protocol with participants securely positioned on the tilt table. After obtaining baseline cardiovascular recordings in the supine position, subjects were passively tilted to 70° and maintained in the upright position for 5 min under continuous cardiovascular monitoring. Heart rate and blood pressure responses were continuously recorded throughout the procedure and subsequently analyzed in accordance with established autonomic function testing guidelines.

For short‐term HRV analysis, participants were positioned in a supine state for a total of 15 min, ensuring physiological stabilization, during which a continuous ECG signal was recorded for 5 min under strictly controlled environmental and clinical conditions. The recorded data underwent HRV quantification through three complementary analytical approaches: time domain analysis (e.g., SDNN and root mean square of successive differences [RMSSD]), frequency domain analysis (e.g., LF and HF components), and nonlinear methods (e.g., Poincaré plots, entropy measures). Cardiac structural and functional assessment was conducted through 2‐dimensional (2D) echocardiography and 24 h Holter monitoring. Standard transthoracic M‐mode, 2D, and Doppler echocardiographic evaluations were performed with patients in the left lateral decubitus position. High‐resolution ultrasound equipment, specifically the Esaote MyLab Seven and Phillips CVxi devices, equipped with a 1.5–4.0 MHz cardiac transducer, were utilized to acquire precise cardiac images. Holter monitoring was performed using the Schiller Medilog FD12 Plus system, enabling continuous ECG recording over a 24‐h period to detect arrhythmias and evaluate HRV during daily activities.

Furthermore, neuroimaging studies were conducted for all patients using a 1.5 T MRI system. Brain MRI sequences are included T1‐weighted, T2‐weighted, fluid‐attenuated inversion recovery (FLAIR), diffusion‐weighted imaging (DWI), apparent diffusion coefficient (ADC), and susceptibility‐weighted imaging (SWI) sequences, allowing detailed visualization of structural and pathological changes in the brain. Similarly, a dedicated 1.5 T cardiac MRI was performed, with a multi‐echo T2 sequence acquired in both short‐ and long‐axis views of the myocardium and ventricles. Quantitative T2 relaxometry measurements were performed on an offline console to assess myocardial tissue characteristics, particularly to detect areas of edema or fibrosis. This comprehensive multimodal approach facilitated an in‐depth evaluation of neurological, autonomic, and cardiac function in patients, providing a robust dataset for subsequent analysis.

### 2.6 Statistical Analysis

The collected data were systematically analyzed utilizing IBM SPSS Statistics software, version 27.0, to ensure rigorous statistical evaluation. Initially, descriptive statistical methods were employed to summarize and characterize the dataset. This included the calculation of central tendency measures such as the mean and median, measures of dispersion such as the standard deviation, and the determination of frequencies and proportions for categorical variables. For the purpose of comparing continuous variables between different study groups, independent samples *t*‐tests were conducted, allowing for the assessment of significant differences in mean values. Furthermore, to examine the strength and direction of associations between selected variables, Kendall's tau correlation coefficient was applied, which is particularly suitable for non‐parametric data or ordinal variables.

All statistical analyses adhered to a predefined threshold for significance, with a *p* value of less than 0.05 (*p* < 0.05) considered statistically significant. This criterion ensured that observed differences or correlations were unlikely to have occurred by chance, thereby reinforcing the validity of the study's findings.

## 3 Results

### 3.1 Participant's Demographics

Table 1 presents an in‐depth demographic and clinical characterization of the study population. A total of 37 patients fulfilling the inclusion criteria were enrolled in this study. Of these, a majority of the participants were male, accounting for 78.4% of the cohort (*n* = 29), whereas female patients comprised 21.6% (*n* = 8), indicating a clear male predominance within the sample. When examining the age distribution of the study population, the largest proportion of patients belonged to the adolescent age group of 11–20 years, representing 54.1% of the cohort. This was followed by the 21–30 years age group, which accounted for 21.6%, whereas younger children aged 0–10 years made up 16.2% of the sample. A smaller percentage of patients (8.1%) fell in the 31–40 years age range, demonstrating that the disease predominantly affects younger individuals. In terms of clinical presentation, neuropsychiatric symptoms were the most commonly observed manifestation, affecting 78.4% of patients. This was followed by hepatic presentations and asymptomatic cases, both contributing equally at 10.8% each. Among the cohort, autonomic complaints were reported in 27.0% of patients. Notably, the majority of these autonomic dysfunction cases (24.3% of the total cohort) were observed within the neuropsychiatric subgroup, indicating a strong association between neuropsychiatric involvement and autonomic disturbances. A small fraction (2.7%) of patients who were initially categorized as asymptomatic also reported autonomic symptoms, with constipation being the most frequently reported autonomic complaint in this subgroup.

**TABLE 1:** Demographic and clinical characters of Wilson's disease patients.

Demographic characteristic	Category	Frequency	Percentage
Gender	Male	29	78.4
	Female	8	21.6
Age (in years)	0–10	6	16.2
	11–20	20	54.1
	21–30	8	21.6
	31–40	3	8.1
Patient history	Neuropsychiatric	29	78.4
	Hepatic	4	10.8
	Asymptomatic	4	10.8
Patients with autonomic complaints	—	10	27.0
	In neuropsychiatric patients	9	24.3
	In asymptomatic patients	1	2.7
Family history	Positive	12	32.4
Treatment history	Penicillamine	36	97.2
	Zinc	22	59.4
	Trientine	1	2.7
	Pallidotomy	1	2.7

**FIGURE 1:**
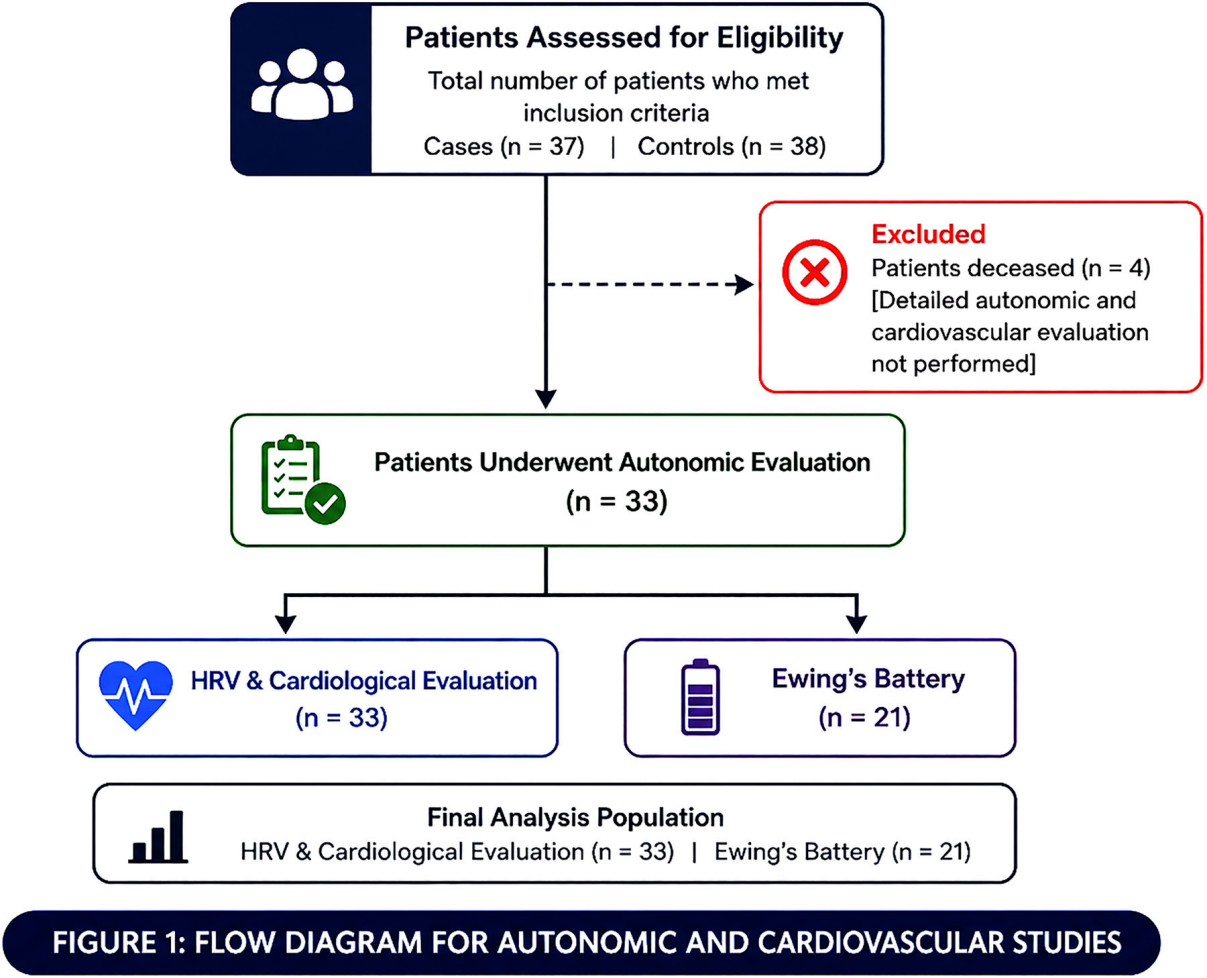
**Study flow diagram for autonomic and cardiovascular evaluation of Wilson's disease patients and control cases (**flowchart is illustrating the participant recruitment, screening, exclusions, and final enrollment in the autonomic and cardiovascular assessment. A total of 37 patients with Wilson's disease and 38 healthy controls fulfilled the inclusion criteria. Four patients died before detailed autonomic and cardiovascular evaluation and were excluded. Consequently, 33 patients underwent heart rate variability [HRV] and cardiac evaluation, of whom 21 additionally completed Ewing's battery of autonomic function tests. These participants constituted the final study population for the respective analyses).

Regarding family history, 32.4% of patients reported a positive family history of the disease, suggesting a significant hereditary component in this population. In terms of treatment patterns, penicillamine emerged as the most commonly prescribed therapeutic agent, administered in 97.2% of cases, reflecting its status as a primary treatment option. Zinc therapy was also widely used, prescribed to 59.4% of patients, whereas trientine and pallidotomy were infrequently utilized, each accounting for only 2.7% of the treatment regimens. Out of the total study population, detailed autonomic function testing, including comprehensive cardiovascular evaluation, was successfully performed in 33 patients. Furthermore, the complete Ewing's battery of cardiovascular reflex tests was fully conducted in 21 patients, as illustrated in Figure 1.

### 3.2 Autonomic Cardiovascular Tests

Table 2 presents a comprehensive comparison of autonomic function test results between patients diagnosed with WD (referred to as the Case group) and healthy control subjects. The evaluation of autonomic function included multiple standardized tests designed to assess both parasympathetic and sympathetic nervous system responses. For the DBT, which primarily assesses parasympathetic activity, the change in heart rate (∆HR in beats per minute) demonstrated a pronounced difference between the two groups. The mean ∆HR in controls was significantly higher than in the WD group, with a mean difference of 10.186 bpm. This difference was statistically significant, with a *t* value of 4.29 and a *p* value less than 0.001, indicating robust evidence of impaired parasympathetic regulation in WD patients. In the DBT (E:I ratio), another marker of parasympathetic function reflecting the ratio of expiration to inspiration HRV, the mean difference between controls and cases was 0.363. This difference was also statistically significant, supported by a *t* value of 4.84 and a *p* value less than 0.001, further corroborating reduced parasympathetic activity in the case group. The HUT test, which assesses both sympathetic and parasympathetic components of cardiovascular autonomic function, showed a significant difference in the 30:15 ratio between controls and cases. The mean difference was 0.373, accompanied by a *t* value of 5.18 and a *p* value less than 0.001, indicating marked autonomic dysfunction in WD patients with impaired cardiovascular reflexes upon positional change. In the Valsalva maneuver, which evaluates both sympathetic and parasympathetic responses through forced exhalation against a closed airway, the Valsalva ratio (VR) exhibited a substantial mean difference of 0.806 between controls and cases. This result was highly significant, with a *t* value of 7.97 and a *p* value <0.001, highlighting severe autonomic impairment in the WD group. Regarding tests specifically designed to evaluate sympathetic function, the isometric hand grip test measured changes in diastolic blood pressure (∆DBP in mm Hg). The mean difference between controls and cases was 4.425 mm Hg, with a *t* value of 2.15 and a *p* value of 0.03, indicating a statistically significant reduction in sympathetic response in WD patients. However, in contrast, the cold pressor test, another sympathetic function assessment involving hand immersion in cold water, did not reveal a statistically significant mean difference in ∆DBP between controls and cases, suggesting that this particular sympathetic reflex may not be as severely affected in WD. All, these findings collectively indicate significant autonomic dysfunction in patients with WD compared to healthy controls. Parasympathetic function, as evaluated by DBTs and VR, was markedly impaired. Similarly, tests involving sympathetic responses, such as the hand grip and HUT tests, showed significant abnormalities. The cold pressor test was the only measure that did not reach statistical significance. Overall, the data underscore a broad spectrum of ANS involvement in WD, with more pronounced deficits in parasympathetic regulation.

**TABLE 2:** Autonomic function tests in Wilson's disease patients and control cases.

Variable	Group case (*n* = 21) control (*n *= 40)	Mean	SD	Mean difference	*t*	*p*
DBT ∆HR (bpm)	Control	22.0811	10.05921	10.186	4.29	<0.001
	Case	11.8947	3.03488			
DBT (E:I)	Control	1.4608	0.3145	0.363	4.84	<0.001
	Case	1.0979	0.11603			
HUT (30:15)	Control	1.4012	0.30386	0.373	5.18	<0.001
	Case	1.0283	0.09825			
VR	Control	1.9181	0.43222	0.806	7.97	<0.001
	Case	1.1116	0.109			
Isometric hand grip ∆DBP (mm Hg)	Control	18.1622	8.1565	4.425	2.15	0.03
	Case	13.7368	5.02043			
Cold pressor ∆DBP (mm Hg)	Control	13.8	4.55	−0.15	−0.12	0.9
	Case	13.65	3.06			

Abbreviations: DBT, deep breathing test; ∆HR, change in heart rate; HUT, head‐up tilt; VR, Valsalva ratio; ∆DBP, change in diastolic blood pressure.

### 3.3 HRV Analysis

Table 3 provides a comprehensive comparison of HRV parameters between patients diagnosed with WD (*n* = 33) and a control group of healthy individuals (*n* = 38). The analysis reveals significant differences in several HRV measures, which reflect variations in ANS regulation between the two groups. First, the standard deviation of R‐R intervals (SDRR), a conventional time‐domain measure reflecting overall HRV and predominantly parasympathetic activity, was found to be significantly higher in the control group compared to the WD patients. Specifically, the mean SDRR value in controls was 55.999 ms, whereas in WD patients, it was 36.781 ms, with a *p* value of 0.01, indicating a statistically significant reduction in overall HRV in the WD group. Second, the average heart rate (AVG rate) showed a notable difference between the two groups. Controls exhibited a significantly lower average heart rate, with a mean value of 72.756 beats per minute (bpm), compared to 81.011 bpm in the WD patients (*p* = 0.002). This suggests an increased resting heart rate in patients, potentially reflecting altered autonomic balance.

**TABLE 3:** Comparison of heart rate variability parameters in Wilson's disease patients and control cases.

Variable	Group case (*n* = 33) controls (*n *= 38)	Mean	SD	Mean difference	*t*	*p*
SDRR	Control	55.999	41.701	19.217	2.51	0.01
	Case	36.781	15.035			
AVG rate	Control	72.756	10.009	−8.254	−3.21	0.002
	Case	81.011	11.469			
RMSSD	Control	57.511	49.031	20.415	1.647	0.1
	Case	37.096	18.356			
LF/HF	Control	1.967	1.019	1.216	3.325	0.001
	Case	0.751	0.615			
SD1	Control	39.696	36.406	13.835	1.655	0.1
	Case	25.861	12.905			
SD2	Control	66.946	30.733	21.009	3.417	0.001
	Case	45.936	18.401			

Abbreviations: AVG rate, average rate; LF/HF ratio, low‐frequency/high‐frequency ratio; RMSSD, root mean square of successive differences between heartbeats; SDRR, standard deviation of R‐R intervals.

Regarding the frequency domain analysis, the low‐frequency to high‐frequency power ratio (LF/HF ratio), an important marker indicating the balance between sympathetic and parasympathetic modulation of heart rate, was significantly higher in the control group compared to WD patients. The mean LF/HF ratio was 1.967 in controls and 0.751 in WD patients, with a highly significant *p* value of 0.001. This finding may indicate an alteration in autonomic modulation and a possible shift in sympathovagal balance in patients with WD. Additionally, the nonlinear HRV parameter SD2, representing long‐term variability and influenced by both sympathetic and parasympathetic inputs, was significantly greater in controls than in WD cases. The mean SD2 was 66.946 ms in controls versus 45.936 ms in patients (*p* = 0.001), further supporting diminished autonomic flexibility in the patient group. However, no significant differences were observed between WD patients and controls in the RMSSD and SD1. Both RMSSD and SD1 are primarily markers of short‐term parasympathetic activity, and their similarity suggests that short‐term vagal modulation may not be significantly altered in WD. All these results indicate that WD is associated with distinct patterns of autonomic dysfunction, characterized by reduced overall HRV (SDRR, SD2), elevated resting heart rate, and a lower LF/HF ratio, pointing toward impaired sympatho‐vagal regulation. These findings suggest underlying physiological differences or disease‐related alterations in autonomic function in WD patients compared to healthy individuals. A known limitation of HRV analysis is that HRV parameters are influenced by multiple physiological and methodological factors, including respiration, circadian rhythm, metabolic status, and emotional stress. These variables could not be fully controlled or systematically analyzed in the present study.

Conventional time‐domain parameters such as SDRR and RMSSD are generally reflective of parasympathetic activity, whereas frequency domain parameters like the LF/HF ratio provide insights into sympatho‐vagal balance. Nonlinear measures SD1 and SD2 represent vagal and sympathetic modulation, respectively, with SD2 being significantly reduced in WD, indicative of compromised long‐term autonomic adaptability.

### 3.4 Correlation Analysis

A detailed correlation analysis was conducted to investigate the relationships between clinico‐demographic factors, biochemical parameters, and autonomic function indicators in patients with WD. Autonomic function was evaluated using Ewing's battery of tests alongside HRV studies, providing a comprehensive assessment of ANS status in this patient population. Notably, the 30:15 ratio obtained during the HUT test demonstrated a significant negative correlation with urinary copper levels, indicating that higher copper excretion is associated with greater impairment in autonomic cardiovascular reflexes. Additionally, the LF/HF ratio, a key marker of sympathovagal balance derived from HRV analysis, exhibited a significant negative correlation with the neurological severity measured by the UWDRS. These findings highlight complex and significant interactions between biochemical abnormalities, neurological impairment, and autonomic dysfunction in WD. The results underscore the importance of further in‐depth studies to elucidate the clinical implications of these associations as well as the underlying pathophysiological mechanisms. Detailed data supporting these correlations are presented in Tables S1 and S2.

### 3.5 2D Echocardiography

Among the various cardiac parameters evaluated in the study, ejection fraction (EF) emerged as the only parameter that demonstrated a statistically significant abnormality in patients (cases) when compared to the control group. The EF values measured in the WD cases were notably lower than those observed in the control population, indicating some degree of subclinical cardiac involvement. However, it is important to emphasize that despite this reduction in EF, none of the patients exhibited clinically significant pump failure or overt HF symptoms during the evaluation.

In addition to EF, other cardiac abnormalities were observed but did not reach statistical significance. Specifically, mild tricuspid regurgitation was detected in five patients, corresponding to a *p* value of 0.4, suggesting that this finding could likely be incidental rather than disease‐related. Furthermore, Grade 1 diastolic dysfunction was identified in three patients, with a *p* value of 0.2, again indicating no statistically significant difference between cases and controls.

These detailed observations are further presented in Table S3, which provides a comprehensive breakdown of the echocardiographic findings. The results suggest that while EF may serve as an early marker of subtle cardiac involvement in WD, gross functional impairment of the heart remains rare in this patient population.

### 3.6 Twenty Four Hours‐Holter Study

**FIGURE 2:**
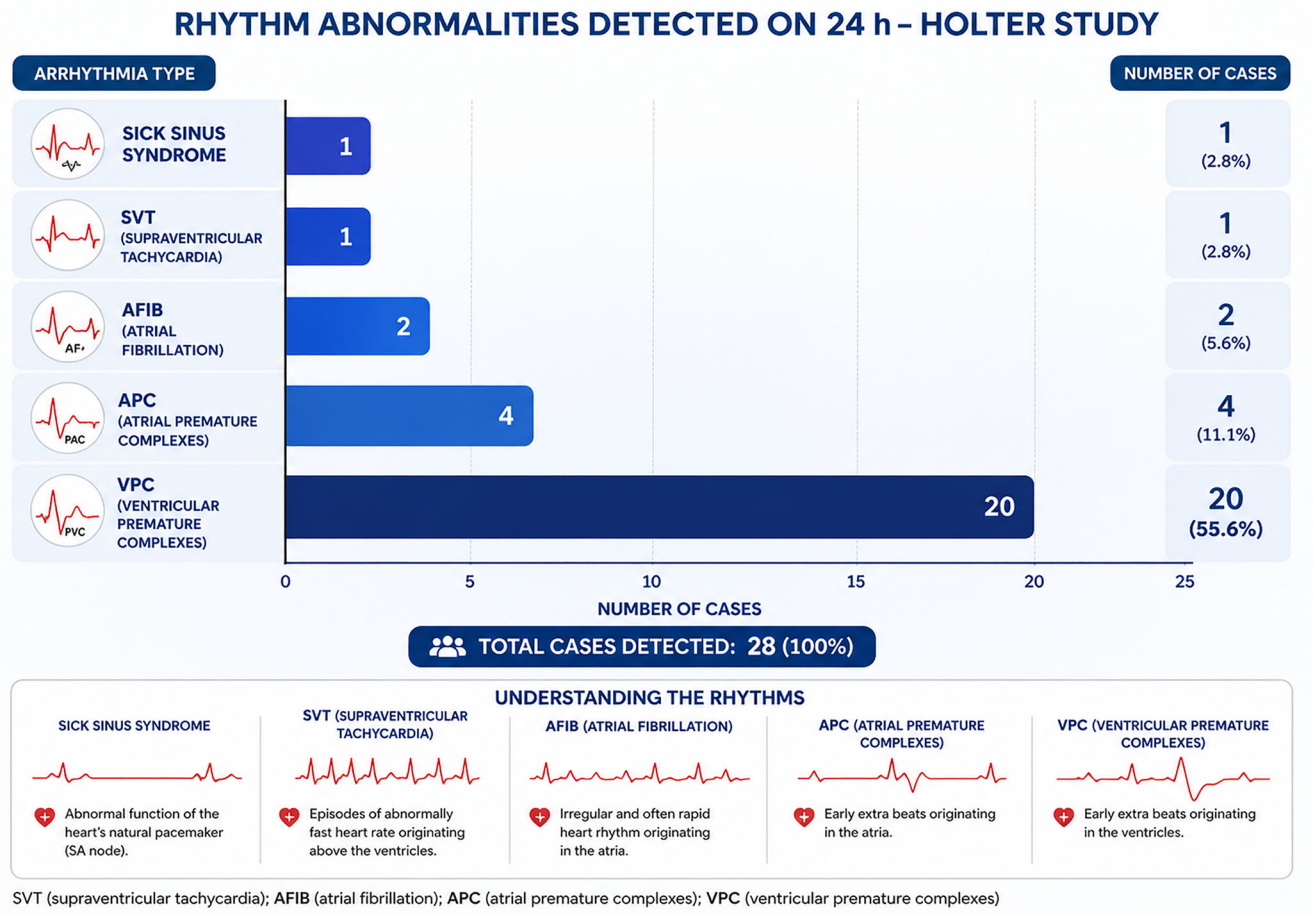
**Distribution of rhythm abnormalities detected on 24‐h Holter monitoring of Wilson's disease patients** (bar chart showing the frequency of rhythm abnormalities identified during 24‐h ambulatory electrocardiographic [Holter] monitoring among study participants. Ventricular premature complexes [VPCs] were the most common abnormality [20 cases; 55.6%], followed by atrial premature complexes [APCs] [4 cases; 11.1%], atrial fibrillation [AF] [2 cases; 5.6%], supraventricular tachycardia [SVT] [1 case; 2.8%], and sick sinus syndrome [1 case; 2.8%]. A total of 28 rhythm abnormalities were detected).

The mean heart rate observed in the patient group was 84 ± 11.46 beats per minute, which was significantly higher compared to the control population, with a *p* value of 0.04, indicating statistical significance. Analysis of 24‐h Holter monitoring revealed various rhythm abnormalities among the patients. The most frequently detected arrhythmia was ventricular premature complexes (VPCs), identified in 20 patients, and followed by atrial premature complexes (APCs) in 4 patients. Additionally, atrial fibrillation (AFib) was observed in two patients, supraventricular tachycardia (SVT) in one patient, and sick sinus syndrome in one patient. Among these, clinically significant rhythm abnormalities, those with potential clinical implications requiring further management, were present in three patients. The distribution and details of these findings are illustrated in Figure 2.

### 3.7 Cardiac MRI

T2* values of the muscular interventricular septum (IVS) showed no significant differences between study groups (*p* > 0.05), indicating comparable myocardial tissue characteristics. Correlation analysis revealed no significant associations between IVS T2* values and serum copper (*p* = 0.14) or ceruloplasmin levels (*p* = 0.1), suggesting that myocardial T2* relaxation times are independent of systemic copper metabolism in this population. Results are detailed in Tables 4, 5, Figure 3a,b and the Supporting Information figure.

**TABLE 4:** Comparison of T2* values between Wilson's disease patients and control cases.

Variable	Group case (*n* = 27) control (*n* = 30)	Mean	SD	Mean difference	*t*	*p*
T2* values of the muscular IVS	Control	36.86	6.38	1.39	0.74	0.46
	Case	38.26	6.32			

**TABLE 5:** Correlation of T2* values with serum copper and ceruloplasmin in Wilson's disease patients.

Correlation	Variable	Mean ± SD	Kendall Tau‐b	*p*
T2* values of muscular IVS (37.866 ± 6.31)	S ceruloplasmin	11.12 ± 6.58	0.21	0.14
	S copper	46.04 ± 18.56	0.22	0.1

FIGURE 3(a) **Correlation between T2 time at the muscular interventricular septum and serum ceruloplasmin concentration of Wilson's disease patients** (scatter plot demonstrating the relationship between T2* magnetic resonance imaging [MRI] values obtained at the muscular interventricular septum [IVS] and serum ceruloplasmin concentrations. Linear regression analysis showed a weak positive correlation that did not reach statistical significance [*r* = 0.25, *R* ^2^ = 0.062, *p* = 0.19], indicating substantial interindividual variability and a poor linear association). (b) **Correlation between T2 time at the muscular interventricular septum and serum copper concentration of Wilson's disease patients** (scatter plot illustrating the association between T2* magnetic resonance imaging [MRI] values measured at the muscular interventricular septum [IVS] and serum copper concentrations. Linear regression demonstrated a weak positive correlation [*r* = 0.329, *R* ^2^ = 0.108] that did not achieve statistical significance [*p* = 0.087], suggesting a trend toward higher serum copper levels with increasing T2* values, although with considerable variability).

**Figure:**
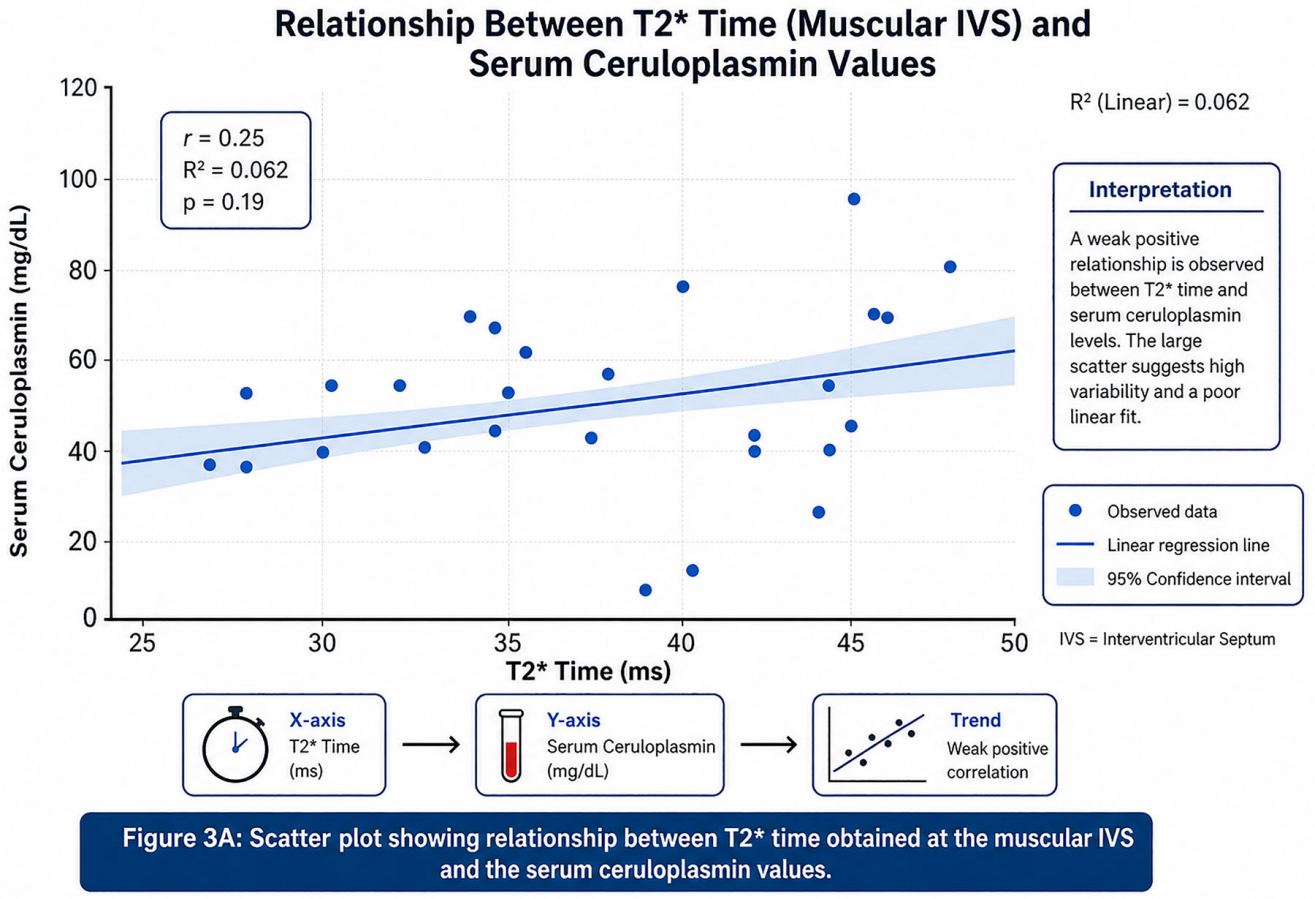

**Figure:**
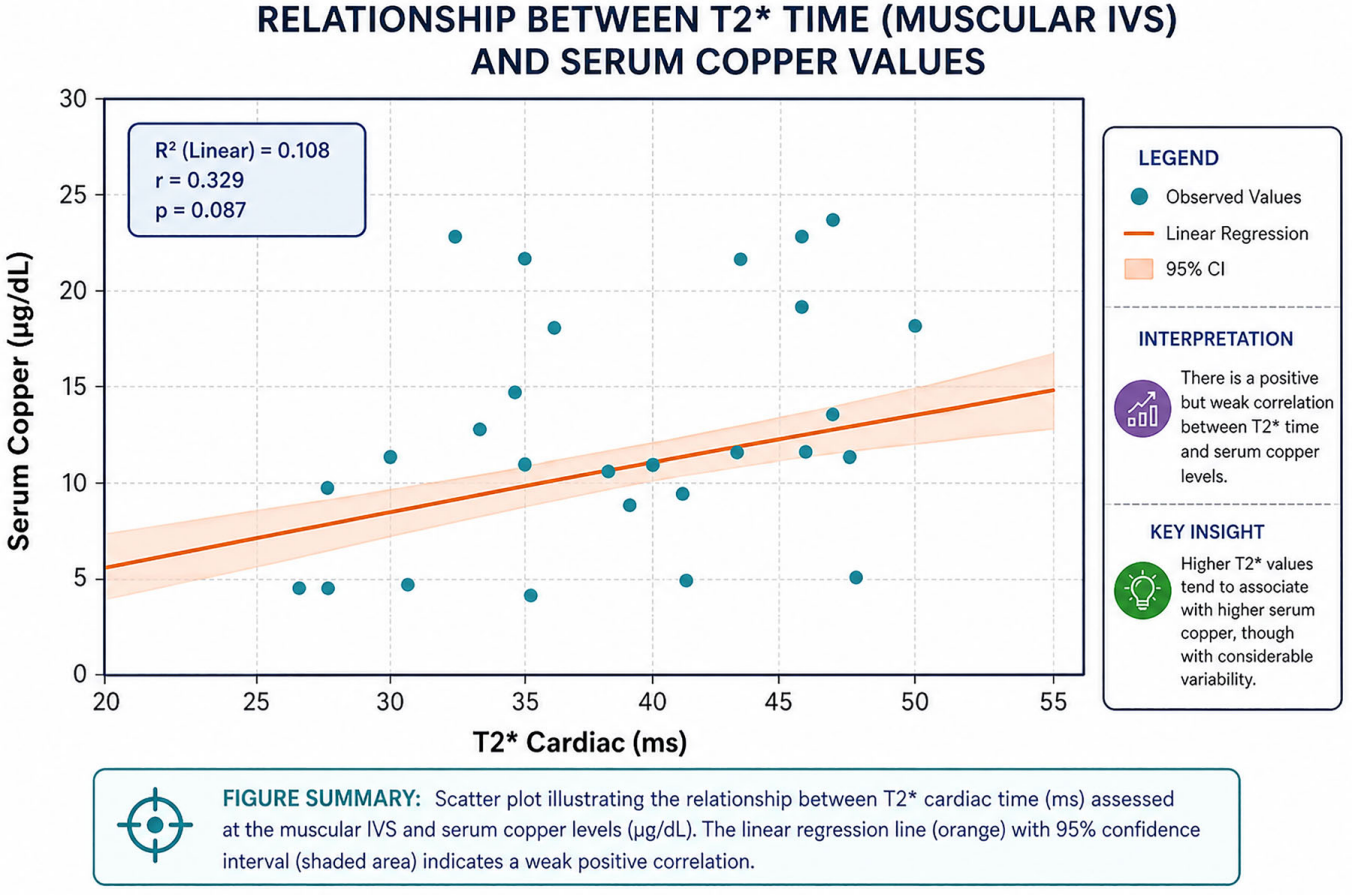

## 4 Discussion

WD is a well‐characterized inherited disorder caused by mutations in the ATP7B gene, which leads to defective copper transport and subsequent copper accumulation in various organs, primarily affecting the liver and the brain (Friedrich et al. 2008). Clinically, the disease most commonly manifests through prominent hepatic dysfunction and a variety of extrapyramidal neurological symptoms, such as tremors, dystonia, and parkinsonism‐like features (Jopowicz and Tarnacka 2023). Recent studies have increasingly demonstrated significant ANS involvement in WD, although longitudinal data on its progression remain limited. Using cardiovascular reflex testing and HRV spectral analysis, the present study identified a wide range of autonomic abnormalities, including elevated resting heart rate, reduced parasympathetic activity, and altered sympathetic responses during autonomic challenges. These findings expand the understanding of WD beyond its classical hepatic and motor manifestations and suggest involvement of central autonomic regulatory pathways. Furthermore, the degree of autonomic dysfunction correlated with neurological severity, indicating that central copper‐mediated neuronal dysfunction may contribute substantially to disease pathophysiology. However, similarities between the autonomic features of WD and Parkinson's disease should not be interpreted as evidence of shared neurodegenerative mechanisms, but rather as distinct manifestations of copper‐induced neuronal injury in WD (Kłysz et al. 2021; Li et al. 2017; Cersosimo and Benarroch 2013; Bandmann et al. 2015; Machado et al. 2006; Coon et al. 2018; Palma and Kaufmann 2018; Jain 2011; Leys et al. 2022; Li et al. 2015). Overall, these outcomes expand our understanding of WD, highlighting its multi‐systemic nature and pointing toward potential avenues for more comprehensive patient management and future research.

The present study comprehensively investigated the demographic profile, clinical characteristics, treatment patterns, and autonomic function in patients with WD. It was observed that a majority of the study population were male, accounting for 78.4% of cases. The age distribution revealed that the highest prevalence of WD occurred in the adolescent and young adult age group, specifically between 11 and 20 years, which constituted 54.1% of the total patients. Clinically, the predominant presenting symptoms were related to neuropsychiatric manifestations, with 78.4% of patients exhibiting such disorders, reflecting the significant neurological involvement in WD. In the context of treatment strategies, the therapeutic regimens primarily comprised penicillamine, prescribed to 97.2% of patients, followed by zinc supplementation in 59.4% of cases. These treatments are consistent with standard management protocols aimed at reducing copper accumulation and alleviating disease progression (Brewer et al. 1998; EASL 2012). A detailed comparison of autonomic function between a cohort of 21 WD patients and a control group of 40 healthy individuals revealed several significant differences. Autonomic function was assessed using standardized tests, including DBT, HUT test (30:15 ratio), Valsalva maneuver (VR), isometric hand grip test (ΔDBP), and cold pressor test (ΔDBP). WD patients showed clear impairments in DBT, HUT (30:15 ratio), and VR, indicating significant autonomic dysfunction when compared to controls. Specifically, isometric hand grip ΔDBP demonstrated substantial differences between groups, whereas cold pressor ΔDBP did not exhibit significant alterations, suggesting selective autonomic pathway involvement.

HRV analysis further supported these findings. The control group exhibited a higher SDRR, indicating greater overall variability in heart rate, whereas the average heart rate was significantly lower than in WD cases. Additionally, the LF/HF ratio was markedly elevated in WD patients, implying an altered balance between sympathetic and parasympathetic modulation, possibly reflecting sympathetic predominance or parasympathetic withdrawal. Furthermore, SD2, a long‐term HRV measure, was significantly greater in controls, reinforcing the notion of reduced autonomic adaptability in WD patients. Interestingly, no significant differences were noted in RMSSD and SD1, which typically reflect short‐term parasympathetic activity. However, the overall pattern of abnormalities strongly suggests predominant parasympathetic involvement in WD‐related autonomic dysfunction (Deguchi et al. 2005; Li et al. 2017; Cheshire et al. 2021; Ooi et al. 2024). Collectively, these results emphasize the presence of significant autonomic dysfunction in patients with WD compared to healthy controls. Early identification of autonomic abnormalities may permit more timely intervention and improved clinical management.

HRV serves as a fundamental and non‐invasive biomarker for assessing ANS function, offering insights into the dynamic interplay between sympathetic and parasympathetic regulation (Deguchi et al. 2005; Li et al. 2017; Cheshire et al. 2021; Ooi et al. 2024). Higher HRV reflects efficient autonomic adaptability, whereas lower HRV is associated with impaired autonomic regulation and several pathological conditions, including cardiovascular disease and chronic stress (Ooi et al. 2024). The present study represents one of the limited investigations conducted in the Indian population examining HRV parameters in WD. In view of the scarcity of region‐specific research, this study highlights the utility of autonomic function assessments, including Ewing's battery of cardiovascular reflex tests, for detecting subclinical autonomic abnormalities in WD patients. Comparative HRV analysis revealed notable differences between WD patients and controls, suggesting autonomic dysfunction as an important yet underrecognized component of WD pathophysiology.

Our study further demonstrated significant associations between disease severity in WD, measured by the UWDRS, and markers of autonomic function (Li et al. 2017). Specifically, a negative correlation was observed between UWDRS scores and the LF/HF ratio of HRV, suggesting worsening autonomic imbalance with increasing disease severity. Additionally, a negative correlation between urinary copper levels and heart rate response during the HUT test indicated that copper accumulation may adversely affect cardiovascular autonomic reflexes. Supporting these findings, Li et al. (2017) reported similar associations between UWDRS scores and autonomic parameters, including DBT (E:I ratio), VR, and handgrip test. These observations strengthen the concept that autonomic dysfunction is an integral component of WD.

Echocardiographic evaluation revealed a statistically significant difference in EF between WD patients and controls, although systolic function remained preserved. Mild tricuspid regurgitation and Grade‐1 diastolic dysfunction were observed in several patients. These findings align with previous studies; Grandis et al. (2017) reported increased prevalence of diastolic dysfunction in WD patients, whereas Buksińska‐Lisik et al. (2019) documented left ventricular hypertrophy and impaired diastolic parameters.

Holter monitoring demonstrated a significantly higher mean heart rate in WD patients. Only one patient developed sick sinus syndrome progressing to complete heart block, whereas others exhibited isolated tachycardia, VPCs, APCs, or atrial fibrillation. Several mechanisms have been proposed to explain copper‐associated cardiac toxicity in WD, including oxidative stress, activation of pro‐inflammatory pathways, genetic polymorphisms affecting antioxidant defenses, and increased apoptosis (Vo et al. 2024; Li et al. 2025; Gromadzka et al. 2024).

Cardiac MRI with analysis of T2* relaxation times was also performed. Although T2* values were lower in WD patients compared to controls, the difference was not statistically significant. MRI‐based approaches have traditionally been used to detect iron overload (Hekmatnia et al. 2010). Kumar et al. (2019) demonstrated correlations between interventricular septal T2* values and liver iron concentration. However, our analysis did not reveal a significant correlation between T2* relaxation times and serum copper levels.

Similarly, Salatzki et al. (2021) reported no significant differences in myocardial T1 and T2 values between WD patients and controls. A plausible explanation may be the diamagnetic nature of copper, which exerts less influence on MRI relaxation times compared with paramagnetic metals such as iron.

## 5 Limitations

The present study has several limitations. Most patients were receiving anti‐copper therapy during evaluation, which may have influenced neurological and autonomic findings. Due to the small sample size and heterogeneity in treatment duration and regimens, subgroup analyses based on treatment exposure could not be performed. Disease severity was assessed using the UWDRS, but severity‐based analyses were limited by insufficient statistical power. Cardiac findings should be interpreted cautiously, as all values remained within normal physiological limits without evidence of overt dysfunction, and no significant correlations were observed with biochemical markers. Additionally, the relatively small sample size, particularly for autonomic testing and HRV analysis, may have reduced the ability to detect subtle differences and increased the risks of Type II and false‐positive errors. Therefore, the findings should be considered exploratory and require validation in larger longitudinal studies.

### 5.1 Future Directions and Clinical Translation

The findings from this study highlight the critical need to incorporate the assessment of autonomic dysfunction into the standard evaluation protocols for patients with WD. Future research should aim to expand upon the initial perceptions gained regarding the prevalence and severity of autonomic dysfunction in WD, as well as its potential implications for patient management and clinical outcomes. Future studies should employ longitudinal designs to track the progression of autonomic dysfunction over time in patients with WD. This approach will help determine the natural history of autonomic impairment and its relationship with disease status and treatment responses. Investigating the underlying mechanisms of autonomic dysfunction in WD is essential. This could involve analyzing the neuroinflammatory processes, copper accumulation in neural tissues, and the impact of both hepatic and extrahepatic manifestations on autonomic pathways. Diverse patient populations should be studied to assess variability in autonomic dysfunction based on genetic, biochemical, and environmental factors. This will enhance our understanding of how demographic variables might influence autonomic involvement in WD. Future research should include comparative studies with other liver diseases known to affect autonomic function, such as cirrhosis, to better understand the unique aspects of autonomic involvement specific to WD. The establishment of specific scoring systems or biomarkers to evaluate and quantify autonomic dysfunction in WD is a crucial area for future exploration. These tools could facilitate early diagnosis and tailored interventions. Investigating the impact of current WD treatments (e.g., chelating agents and zinc therapy) on autonomous function will be necessary to determine if enhancing copper metabolism can also alleviate autonomic dysfunction. Moreover, potential novel therapeutic strategies targeting autonomic regulation could be developed and tested.

## 6 Conclusion

This study provides important insights into autonomic dysfunction in patients with WD, a frequently under‐recognized aspect of the disorder. Significant differences in autonomic function tests were observed between WD patients and healthy controls, particularly in parameters reflecting parasympathetic activity, suggesting that ANS involvement may contribute substantially to the disease process beyond its classical hepatic and neuropsychiatric manifestations. Despite limitations such as the small sample size and the potential influence of motor deficits on autonomic testing, the findings emphasize the importance of routine autonomic evaluation in WD. The study also supports the incorporation of Holter monitoring and 2D echocardiography into early clinical assessment to detect subclinical cardiac dysfunction. Further large‐scale studies are required to validate these findings, clarify underlying pathophysiological mechanisms, and explore advanced imaging markers and therapeutic strategies for autonomic and cardiac involvement in WD.

## Author Contributions

**Sabyasachi Pattanayak**: conceptualization, methodology, writing – review and editing. **Niraj Kumar Srivastava**: conceptualization, methodology, writing – review and editing. **Anand Kumar**: conceptualization, methodology, writing – review and editing. **Deepika Joshi**: conceptualization, methodology, project administration, supervision, writing – review and editing. **Atanu Roy**: methodology, investigations, resources. **Sanjeev Kumar Singh**: methodology, investigations, resources. **Rajniti Prasad**: resources, validation, supervision. **Suyash Tripathi**: methodology, investigations, resources. **Ashish Verma**: methodology, investigations, resources. **Ritu Ojha**: methodology, investigations, resources. **Varun Kumar Singh**: resources, data curation, supervision. **Abhishek Pathak**: resources, data curation, supervision. **Rameshwar Nath Chaurasia**: resources, data curation, supervision. **Vijaya Nath Mishra**: resources, data curation, supervision. **Ibrahim Hussain**: resources, data curation, supervision.

## Funding

The authors have nothing to report.

## Ethics Statement

The research was conducted in strict adherence to established ethical principles and guidelines governing human subject research. Prior to initiating the study, ethical approval was obtained from the Institutional Ethics Committee, ensuring full compliance with institutional and national regulations governing research conduct (Approval reference: Dean/2021/ECI/2896). In accordance with the ethical committee's recommendations, we ensured that all study participants, including both patients and control subjects, were provided with comprehensive information regarding the study's purpose, procedures, potential risks, and benefits. Written informed consent was diligently obtained from each participant before their enrolment in the study, thereby upholding the highest standards of autonomy, transparency, and ethical responsibility throughout the research process.

## Consent

All participants (or their legal guardians, where applicable) provided informed consent for the publication of the data, images, and findings included in this manuscript. The authors affirm that consent was obtained in accordance with institutional and ethical guidelines, ensuring that participants were fully informed about the purpose of publication, the type of information to be shared, and their right to confidentiality. No identifying information has been disclosed that would compromise participant privacy.

## Conflicts of Interest

The authors declare no conflicts of interest.

## Supporting information




**Supporting Information**:Representative cardiac magnetic resonance imaging of Wilson's disease patients [Representative cardiac magnetic resonance imaging (CMR) T2* parametric map obtained using a multi‐echo gradient‐echo sequence for myocardial copper assessment. The image demonstrates color‐coded signal intensity distribution within the myocardium with regions of interest positioned over the interventricular septum for T2* analysis].

**Supplementary Tables**: brb371624‐sup‐0001‐Tables.docx

## Data Availability

All data generated or analyzed during the study are included with the article and Supporting Information.

## References

[brb371624-bib-0001] Ala, A. , A. P. Walker , K. Ashkan , J. S. Dooley , and M. L. Schilsky . 2007. “Wilson's Disease.” Lancet 369: 397–408. 10.1016/S0140-6736(07)60196-2. 17276780

[brb371624-bib-0002] Bandmann, O. , K. H. Weiss , and S. G. Kaler . 2015. “Wilson's Disease and Other Neurological Copper Disorders.” Lancet Neurology 14, no. 1: 103–113. 10.1016/S1474-4422(14)70190-5. 25496901 PMC4336199

[brb371624-bib-0003] Bhattacharya, K. , M. Velickovic , M. Schilsky , and H. Kaufmann . 2002. “Autonomic Cardiovascular Reflexes in Wilson's Disease.” Clinical Autonomic Research 12: 190–192. 12269552 10.1007/s10286-002-0017-y

[brb371624-bib-0004] Biswas, S. , N. Paul , and S. K. Das . 2017. “Nonmotor Manifestations of Wilson's Disease.” International Review of Neurobiology 134: 1443–1459. 28805579 10.1016/bs.irn.2017.04.010

[brb371624-bib-0005] Brewer, G. J. , R. D. Dick , V. D. Johnson , J. A. Brunberg , K. J. Kluin , and J. K. Fink . 1998. “Treatment of Wilson's Disease With Zinc: XV Long‐Term Follow‐Up Studies.” Journal of Laboratory and Clinical Medicine 132: 264–278. 10.1016/S0022-2143(98)90039-7. 9794697

[brb371624-bib-0006] Buksińska‐Lisik, M. , T. Litwin , T. Pasierski , and A. Członkowska . 2019. “Cardiac Assessment in Wilson's Disease Patients Based on Electrocardiography and Echocardiography Examination.” Archives of Medical Science 15: 857–864. 10.5114/aoms.2017.69728. 31360180 PMC6657248

[brb371624-bib-0007] Cersosimo, M. G. , and E. E. Benarroch . 2013. “Central Control of Autonomic Function and Involvement in Neurodegenerative Disorders.” Handbook of Clinical Neurology 117: 45–57. 24095115 10.1016/B978-0-444-53491-0.00005-5

[brb371624-bib-0008] Cheshire, W. P. , R. Freeman , C. H. Gibbons , et al. 2021. “Electrodiagnostic Assessment of the Autonomic Nervous System: A Consensus Statement Endorsed by the American Autonomic Society, American Academy of Neurology, and the International Federation of Clinical Neurophysiology.” Clinical Neurophysiology 132: 666–682. 10.1016/j.clinph.2020.11.024. 33419664

[brb371624-bib-0009] Chu, E. C. , N. S. Chu , and C. C. Huang . 1997. “Autonomic Involvement in Wilson's Disease: A Study of Sympathetic Skin Response and RR Interval Variation.” Journal of the Neurological Sciences 149: 131–137. 10.1016/S0022-510X(97)05365-3. 9171319

[brb371624-bib-0010] Coon, E. A. , J. K. Cutsforth‐Gregory , and E. E. Benarroch . 2018. “Neuropathology of Autonomic Dysfunction in Synucleinopathies.” Movement Disorders 33, no. 3: 349–358. 10.1002/mds.27186. 29297596

[brb371624-bib-0011] Deguchi, K. , I. Sasaki , T. Touge , et al. 2005. “Improvement of Cardiovascular Autonomic Dysfunction Following Anti–Copper Therapy in Wilson's Disease.” Journal of Neurology 252: 495–497. 10.1007/s00415-005-0674-6. 15726258

[brb371624-bib-0012] Deguti, M. M. , J. Genschel , E. L. R. Cancado , et al. 2004. “Wilson Disease: Novel Mutations in the ATP7B Gene and Clinical Correlation in Brazilian Patients.” Human Mutation 23: 398. 10.1002/humu.9227. 15024742

[brb371624-bib-0013] European Association for the Study of the Liver . 2012. “EASL Clinical Practice Guidelines: Wilson's Disease.” Journal of Hepatology 56: 671–685. 10.1016/j.jhep.2011.11.007. 22340672

[brb371624-bib-0014] Friedrich, C. , H. Rüdiger , C. Schmidt , et al. 2008. “Baroreflex Sensitivity and Power Spectral Analysis in Different Extrapyramidal Syndromes.” Journal of Neural Transmission 115: 1527–1536. 10.1007/s00702-008-0127-3. 18806923

[brb371624-bib-0015] Grandis, D. J. , G. Nah , I. R. Whitman , et al. 2017. “Wilson's Disease and Cardiac Myopathy.” American Journal of Cardiology 120: 2056–2060. 10.1016/j.amjcard.2017.08.025. 28947309

[brb371624-bib-0016] Granowetter, L. 1995. “Ewingʼs Sarcoma and Extracranial Peripheral Neuroectodermal Tumors.” Current Opinion in Oncology 7: 355–360. 10.1097/00001622-199507000-00011. 7578384

[brb371624-bib-0017] Gromadzka, G. , A. Antos , Z. Sorysz , and T. Litwin . 2024. “Psychiatric Symptoms in Wilson's Disease—Consequence of ATP7B Gene Mutations or Just Coincidence?—Possible Causal Cascades and Molecular Pathways.” International Journal of Molecular Sciences 25: 12354. 10.3390/ijms252212354. 39596417 PMC11595239

[brb371624-bib-0018] Hekmatnia, A. , A. R. Radmard , A. A. Rahmani , A. Adibi , and H. Khademi . 2010. “Magnetic Resonance Imaging Signal Reduction May Precede Volume Loss in the Pituitary Gland of Transfusion‐Dependent Beta‐Thalassemic Patients.” Acta Radiologica 51: 71–77. 10.3109/02841850903292743. 20001472

[brb371624-bib-0019] Jain, S. 2011. “Multi‐Organ Autonomic Dysfunction in Parkinson Disease.” Parkinsonism & Related Disorders 17, no. 2: 77–83. 10.1016/j.parkreldis.2010.08.022. 20851033 PMC3021587

[brb371624-bib-0020] Jopowicz, A. , and B. Tarnacka . 2023. “Neurological Wilson's Disease Signs—Hepatic Encephalopathy or Copper Toxicosis?” Diagnostics 13: 893. 10.3390/diagnostics13050893. 36900037 PMC10001333

[brb371624-bib-0021] Joshi, S. P. , M. Thomas , S. Yoganathan , S. Danda , M. Chandran , and A. Jasper . 2023. “Clinico‐Etiological Spectrum and Functional Outcomes of Children With Pre‐Status Dystonicus and Status Dystonicus (SD): A Descriptive Study.” Annals of Indian Academy of Neurology 26: 268–274. 10.4103/aian.aian_660_22. 37538432 PMC10394458

[brb371624-bib-0022] Kłysz, B. , J. Bembenek , M. Skowrońska , A. Członkowska , and I. Kurkowska‐Jastrzębska . 2021. “Autonomic Nervous System Dysfunction in Wilson's Disease—A Systematic Literature Review.” Autonomic Neuroscience 236: 102890. 34656966 10.1016/j.autneu.2021.102890

[brb371624-bib-0036] Kumar, S. 2005. “Severe Autonomic Dysfunction as a Presenting Feature of Wilson's Disease.” Journal of Postgraduate Medicine 51: 75–76. 15793350

[brb371624-bib-0037] Kumar, I. , et al. 2019. “Cardiac T2* MRI Analysis of Membranous Interventricular Septum in Pediatric Thalassemia.” Indian J Radiol Imaging 29: 33–39. 31000939 10.4103/ijri.IJRI_395_18 PMC6467041

[brb371624-bib-0024] Lafhal, K. , E.‐S. Sabir , A. Hakmaoui , et al. 2023. “Clinical, Biochemical and Molecular Characterization of Wilson's Disease in Moroccan Patients.” Molecular Genetics and Metabolism Reports 36: 100984. 10.1016/j.ymgmr.2023.100984. 37323222 PMC10267639

[brb371624-bib-0025] Leys, F. , G. K. Wenning , and A. Fanciulli . 2022. “The Role of Cardiovascular Autonomic Failure in the Differential Diagnosis of α‐Synucleinopathies.” Neurological Sciences 43, no. 1: 187–198. 10.1007/s10072-021-05746-6. 34817726 PMC8724069

[brb371624-bib-0026] Li, K. , C. Lindauer , R. Haase , et al. 2017. “Autonomic Dysfunction in Wilson's Disease: A Comprehensive Evaluation During a 3‐Year Follow Up.” Frontiers in Physiology 8: 778. 10.3389/fphys.2017.00778. 29066979 PMC5641386

[brb371624-bib-0027] Li, K. , H. Reichmann , and T. Ziemssen . 2015. “Recognition and Treatment of Autonomic Disturbances in Parkinson's Disease.” Expert Review of Neurotherapeutics 15: 1189–1203. 10.1586/14737175.2015.1095093. 26416396

[brb371624-bib-0028] Li, L. , L. Lv , Z. Wang , et al. 2025. “From Copper Homeostasis to Cuproptosis: A New Perspective on CNS Immune Regulation and Neurodegenerative Diseases.” Frontiers in Neurology 16: 1581045. 10.3389/fneur.2025.1581045. 40510202 PMC12158703

[brb371624-bib-0029] Machado, A. , H. Fen Chien , M. Mitiko Deguti , et al. 2006. “Neurological Manifestations in Wilson's Disease: Report of 119 Cases.” Movement Disorders 21, no. 12: 2192–2196. 10.1002/mds.21170. 17078070

[brb371624-bib-0030] Meenakshi‐Sundaram, S. , A. B. Taly , V. Kamath , G. R. Arunodaya , S. Rao , and H. S. Swamy . 2002. “Autonomic Dysfunction in Wilson's Disease—A Clinical and Electrophysiological Study.” Clinical Autonomic Research 12: 185–189. 10.1007/s10286-002-0038-6. 12269551

[brb371624-bib-0031] Ooi, J. H. , R. Lim , H. Seng , et al. 2024. “Non‐Invasive Parameters of Autonomic Function Using Beat‐to‐Beat Cardiovascular Variations and Arterial Stiffness in Hypertensive Individuals: A Systematic Review.” Biomedical Engineering Online 23: 23. 10.1186/s12938-024-01202-6. 38378540 PMC10880234

[brb371624-bib-0032] Palma, J. A. , and H. Kaufmann . 2018 Mar. “Treatment of Autonomic Dysfunction in Parkinson Disease and Other Synucleinopathies.” Movement Disorders 33, no. 3: 372–390. 10.1002/mds.27344. 29508455 PMC5844369

[brb371624-bib-0033] Salatzki, J. , I. Mohr , J. Heins , et al. 2021. “The Impact of Wilson Disease on Myocardial Tissue and Function: A Cardiovascular Magnetic Resonance Study.” Journal of Cardiovascular Magnetic Resonance 23: 84. 10.1186/s12968-021-00760-1. 34162411 PMC8223377

[brb371624-bib-0034] Vo, T. T. T. , T.‐Y. Peng , T. H. Nguyen , et al. 2024. “The Crosstalk Between Copper‐Induced Oxidative Stress and Cuproptosis: A Novel Potential Anticancer Paradigm.” Cell Communication and Signaling 22: 353. 10.1186/s12964-024-01726-3. 38970072 PMC11225285

[brb371624-bib-0035] Yuan, X. Z. , R. M. Yang , and X. P. Wang . 2021. “Management Perspective of Wilson's Disease: Early Diagnosis and Individualized Therapy.” Current Neuropharmacology 19: 465–485. 32351182 10.2174/1570159X18666200429233517 PMC8206458

