# Supplementary material for: A Comprehensive and Critical Evaluation of Autonomic Nervous System Impairment in Patients with Wilson Disease: A Cross‐Sectional Study
Source: Brain Behav. 2026 Aug 10;16(8):e71624. doi: 10.1002/brb3.71624 (PMC13458031; doi:10.1002/brb3.71624)
Supplement: Supplementary file 1 [file BRB3-16-e71624-s001.docx]

**SUPPLEMENTARY TABLES**

**TABLE 1: CORRELATION COEFFICENTS FOR AUTONOMIC PARAMETERS (EWING’S BATTERY) AND CLINICO-DEMOGRAPHIC VARIABLES.**

|  | DBT: ∆HR | DBT (E: I) | VR | CPT  (∆DBP) | HG  (∆DBP) | HUT (30:15) |
| --- | --- | --- | --- | --- | --- | --- |
| Duration of illness | 0.17 | -0.13 | 0.06 | 0.04 | 0.04 | -0.19 |
| UWDRS | 0.21 | -0.25 | -0.31 | -0.18 | 0.03 | -0.15* |
| S.Ceruloplasmin | -0.21 | 0.1 | 0.17 | -0.12 | 0.09 | 0.27 |
| S. Copper | -0.02 | 0.32 | 0.03 | 0.07 | 0.15 | 0.03 |
| Urinary copper | -0.12 | -0.18 | -0.17 | -0.05 | -0.14 | -0.36 |

DBT (deep breathing test), VR (Valsalva ratio), isometric hand grip, head up tilt (HUT), ∆HR (change in heart rate), ∆DBP (change in diastolic blood pressure UWDRS (Unified Wilson’s Disease Rating Score

*Sign indicates a p-value<0.05

**TABLE 2: CORRELATION COEFFICENTS FOR AUTONOMIC PARAMETERS(HRV) AND CLINICO-DEMOGRAPHIC VARIABLES**

|  | RESTING  HR | SDRR | RMSSD | LF/HF | SD1 | SD2 |
| --- | --- | --- | --- | --- | --- | --- |
| Duration of illness | 0.18 | -0.01 | -0.13 | 0.05 | -0.13 | 0.09 |
| UWDRS | -0.09 | -0.2 | 0.01 | -0.39 | 0.02 | -0.21 |
| S.Ceruloplasmin | -0.07 | 0.24 | 0.19 | 0.1 | 0.19 | 0.22 |
| S.Copper | 0.03 | 0.23 | 0.23 | -0.15 | 0.22 | 0.22 |
| Urinary copper | -0.09 | -0.11 | -0.16 | 0.02* | -0.11 | -0.03 |

LF/HF ratio (low frequency/high frequency ratio), RMSSD (root mean square of successive differences between heartbeats), SDRR (standard deviation of R-R intervals) UWDRS (Unified Wilson’s Disease Rating Score)

*Sign indicates a p-value<0.05

**TABLE 3: 2D ECHO PARAMETERS IN PATIENTS IN THE PATIENTS AND THE CONTROLS**

| Variable | Group | Mean | SD | Mean diff | t | p |
| --- | --- | --- | --- | --- | --- | --- |
| RSVP | Control | 17.72 | 2.27 | 0.28 | 0.45 | 0.65 |
|  | Case | 18 | 2.2 |  |  |  |
| LV function | Control | 64.5 | 3.44 | -2.47 | 02.93 | 0.005 |
|  | Case | 62.02 | 3.49 |  |  |  |
| E/A | Control | 1.1 | 0.24 | 0.06 | 1.06 | 0.29 |
|  | Case | 1.16 | 0.24 |  |  |  |
| PHT | Control | 50.02 | 5.46 | 1.97 | 1.07 | 0.3 |
|  | Case | 51.99 | 5.36 |  |  |  |
| MVA | Control | 4.38 | 0.39 | -0.13 | -1.04 | 0.3 |
|  | Case | 4.24 | 0.36 |  |  |  |
| Medial e | Control | 0.145 | 0.05 | 0.0018 | 0.09 | 0.9 |
|  | Case | 0.146 | 0.06 |  |  |  |
| Lateral e | Control | 0.17 | 0.02 | 0.0019 | 0.25 | 0.8 |
|  | Case | 0.172 | 0.02 |  |  |  |

RVSP(right ventricle systolic pressure);LV function (left ventricle function);E/A(early wave to late wave ratio);PHT(pulmonary half time);MVA(mitral valve area)
